# Polyalanine Expansion in PABPN1 Alters the Structure and Dynamics of Its Nuclear Aggregates in Differentiated Muscle Cells

**DOI:** 10.1096/fj.202501097R

**Published:** 2025-06-24

**Authors:** Sander D. Mallon, Erik Bos, Vahid Sheikhhassani, Milad Shademan, Lenard M. Voortman, Alireza Mashaghi, Thomas H. Sharp, Vered Raz

**Affiliations:** ^1^ Department of Human Genetics Leiden University Medical Center Leiden the Netherlands; ^2^ Department of Cell and Chemical Biology Leiden University Medical Center Leiden the Netherlands; ^3^ Medical Systems Biophysics and Bioengineering, Division of Systems Pharmacology and Pharmacy Leiden Academic Centre for Drug Research, Leiden University Leiden the Netherlands; ^4^ School of Biochemistry, University of Bristol Bristol UK

**Keywords:** aggregates structure, imaging, muscle, OPMD, protein aggregates

## Abstract

Intracellular protein aggregation is a hallmark of aging and contributes to pathology in some age‐associated diseases. In hereditary adult‐onset neuromuscular diseases (NMDs), protein aggregates play a key role in disease onset and progression. The wild‐type Poly(A) binding protein nuclear 1 (PABPN1) forms benign nuclear aggregates, whereas a short trinucleotide expansion leads to the formation of pathogenic aggregates, a hallmark of Oculopharyngeal Muscular Dystrophy (OPMD). In OPMD, the mutant PABPN1 causes skeletal muscle weakness. So far, the structural differences between benign and pathogenic protein aggregates and their effects on muscle cell biology remain poorly understood. We employed an array of advanced imaging modalities to explore the morphological differences between nuclear aggregates formed by non‐pathogenic and pathogenic PABPN1 variants. Through analyses spanning micro‐ to nanoscale, we identified distinct structural features of aggregates formed by wild‐type and expanded PABPN1. We demonstrate that these differences were more pronounced in differentiated muscle cells compared to proliferating cells. We further linked the structural features of PABPN1 aggregates to muscle cell biology, namely alterations in mitochondrial function and proteasomal activity. Our findings provide new insights into the structural distinctions between pathogenic and non‐pathogenic aggregates and their implications for cellular dysfunction in NMDs.

## Graphical abstract

The accumulation of aggregation‐prone proteins is a hallmark of aging cells. Pathogenic forms of these aggregates are associated with neurodegenerative, neuromuscular, and muscular diseases, yet the differences between pathogenic and non‐pathogenic aggregates remain unclear. In this study, we created an inducible cell model to investigate the structural and molecular properties of nuclear protein aggregates in both undifferentiated and differentiated muscle cells. Through imaging at micro‐ to nanoscale resolutions, we identified key molecular and structural distinctions between non‐pathogenic and pathogenic protein forms. We also linked these structural features to cellular function, providing new insights into the functional differences between pathogenic and non‐pathogenic aggregates.

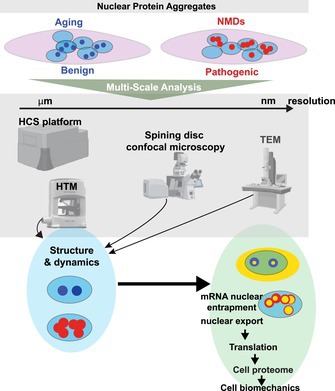

## 1 Introduction

Misfolded proteins are normally degraded by the cell's quality control systems, such as proteasomes and autophagy pathways. Loss of protein homeostasis is a natural process that leads to protein aggregation during aging [1, 2, 3]. Over time, the efficiency of proteostatic mechanisms declines, leading to an increase in protein misfolding and aggregation [4]. However, in diseases such as neurodegeneration and some forms of muscular dystrophy, mutations cause proteins to resist degradation [5]. These mutated proteins accumulate and form pathogenic aggregates that contribute to cell dysfunction. Pathogenic protein aggregates can be cytoplasmic, as seen in Parkinson's disease and Alzheimer's disease, or nuclear as found in polyglutamine repeat expansion disorders, including Huntington's disease (HD) and Spinocerebellar ataxia diseases (SCAs), or in polyalanine repeat expansion disorders like Oculopharyngeal muscular dystrophy (OPMD) [6].

OPMD is caused by a short expansion mutation in the gene encoding poly(A)‐binding protein nuclear 1 (PABPN1) [7]. Wildtype PABPN1 has a repeat of 10 alanine residues at its N‐terminus, but the expansion results in the mutant protein containing 11–18 alanine residues. PABPN1 is ubiquitously expressed and essential in all eukaryotic cells, yet OPMD symptoms are restricted to skeletal muscle, where aggregation of PABPN1 in myonuclei serves as the defining pathological hallmark [8, 9]. Both wild‐type and expanded PABPN1 are prone to aggregate, but only aggregates of the mutant protein are pathogenic [10]. Molecular and structural studies showed that PABPN1 aggregation requires the coiled‐coiled domain [8, 11, 12], whereas the length of alanine expansion does not consistently correlate with symptom severity [13, 14, 15]. Discriminating between structural features of the pathogenic PABPN1 aggregates and the non‐pathogenic form has been addressed in previous studies using mitotic, non‐muscle cells [8, 9, 16, 17], but has not captured the nuances of aggregation in post‐mitotic cells and its effect on muscle cell biology [18]. Understanding the differences between pathogenic and non‐pathogenic aggregated proteins is crucial for deciphering how protein aggregation in muscle disease differs from natural muscle aging. This knowledge can inform the development of targeted, disease‐specific therapies.

In this study, we investigated the structural distinctions between pathogenic and non‐pathogenic PABPN1 protein variants using a human muscle cell model. We investigated the A16 expansion, which is the most prominent in the OPMD Dutch cohort [14]. We generated stable muscle cells expressing wild‐type PABPN1 (Ala10) or the pathogenic allele (Ala16) under the tetracycline‐inducible promoter to bypass the reported toxic effect of PABPN1 in mitotic cells [19]. We compared the structure of pathogenic and non‐pathogenic PABPN1 aggregates using four imaging modalities, ranging from micrometer to nanometer resolution, and linked aggregate structure to muscle cell biology, providing new insights into the structure‐pathogenesis relationship of PABPN1.

## 2 Materials and Methods

### 2.1 PABPN1 Constructs and Lentivirus Production

The full‐length PABPN1 wild type (Ala10) or the expanded PABPN1 (Ala16) fused to YFP, as previously described [16], was cloned into the pCW57‐MCS1‐2A‐MCS2 doxycycline (Dox) inducible lentiviral vector (Addgene plasmid #71782). Cloning was confirmed by Sanger sequencing. Lentivirus production was performed as detailed in [20]. Lentivirus particle titers were determined in HeLa cells.

### 2.2 Cell Culture

The 2417 immortal human muscle cells [21] were propagated in proliferation medium consisting of F10 (Gibco) supplemented with 15% FCS, 1 ng/mL bFGF, 10 ng/mL EGF, and 0.4 μg/mL Dexamethasone. Cells were seeded at 40%–50% confluence and reseeded after reaching 80% confluence to prevent spontaneous differentiation. Cell differentiation was induced at high confluency (85%–95%) in DMEM supplemented with 2% horse serum for 3–5 days. Cells were transduced with lentiviruses encoding Ala10‐YFP or Ala16‐YFP, and stable cell cultures were created using puromycin selection. Induction of the PABPN1‐YFP transgene was carried out with 4 mg/mL doxycycline hyclate (D5207, Sigma Aldrich). For high content screening (HCS), cells were seeded in a Nunc 96‐well plate; for live cell confocal microscopy, cells were seeded in either a μ‐Slide 15 Well 3D ibiTreat (81 506, IBIDI) or a μ‐Slide 8 Well high ibiTreat (80 806, IBIDI) slide. For electron microscopy, cells were seeded in μ‐Dish 35 mm, high Grid‐500 ibiTreat (81 166, IBIDI) dishes. For refractive index imaging, cells were cultured on a μ‐Dish 35 mm, low ibiTreat (80 136, IBIDI) dish. Cell cultures were treated with 0.5 μM epoxomicin (Sigma‐Aldrich #134381–21‐8) for 6 h; Leptomycin B (LMB) 20 nM for 4 h; or 20 μg/mL cycloheximide (Sigma‐Aldrich #01810) for 1 h. Cells were incubated in the growth medium.

### 2.3 Protein Extraction and Western Blotting

Proteins were lysed from cells using RIPA buffer (20 mM Tris, pH 7.4, 150 mM NaCl, 5 mM EDTA, 1% NP40, 5% glycerol, and 1 mM DTT with protease inhibitor cocktail). After sonication and centrifugation (1 min, 13,000 ***g***, at 4°C), the supernatant containing the soluble proteins was transferred to a new tube, and the pellet containing the insoluble proteins was washed once in PBS, dissolved in loading buffer, sonicated, and spun down prior to heat inactivation. Protein aliquots were separated on 10% SDS‐PAGE. Western blotting was carried out with a PVDF membrane. Bulk proteins were visualized with the No‐Stain Protein Labeling Reagent (#A44717, ThermoFisher) and imaged using the iBright Imaging System (ThermoFisher). The membrane was blocked with 5% dried milk powder (T145.2, Carl Roth), primary antibody incubation was carried out at 4° overnight, and secondary antibody incubation at RT for 1 h. Antibodies are listed in Table S1. Detection of fluorescent signals was performed using an Odyssey CLx Infrared Imaging System (LiCOR, NE, USA).

Western blot quantification was performed in ImageJ to assess protein abundance. Values were corrected for background and normalized to loading controls. Normalization was performed using both the No‐Stain and housekeeping protein signal. All Western blotting experiments were performed in six biological replicates.

### 2.4 Immunohistochemistry

Insoluble PABPN1 was detected after 1 M KCl pre‐treatment for 15 min. Immunohistochemistry with or without KCl pre‐treatment was performed using standard procedures: fixation (2% formaldehyde in PBS) for 5 min, permeabilization (1% Triton in PBS) for 10 min, PBS washing, incubation with primary antibodies for 1 h at RT, PBS washing, incubation with secondary antibodies and DAPI (4′,6‐Diamidino‐2‐phenylindole dihydrochloride) for 30 min, and PBS washing. Cells were kept in PBS during imaging. Antibodies are listed in Table S1.

### 2.5 Cellular Assays

Mitochondrial activity assays were performed using Tetramethylrhodamine methyl ester perchlorate (TMRM). Cell cultures in 96‐well plates were washed with PBS and incubated with a staining solution (5 nM TMRM [Sigma‐Aldrich #115532–50‐8] and Hoechst) diluted in growth media and incubated for 45 min. After two PBS washes, cells were kept in differentiation media during imaging.

Protein synthesis was assessed using the Protein Synthesis Assay Kit (Cayman Chemicals #601100) according to the manufacturer's protocol, with the following modifications: azido‐O‐propargyl‐puromycin (OPP)‐488 was replaced with OPP‐555 (Vector Laboratories, #CCT‐1494). For the negative control, a 30‐min pre‐incubation with 20 μM cycloheximide was used. Hoechst (33 342 ThermoFisher, final concentration 1 μg/mL) was added after fixation.

Poly(A) + RNA was visualized by RNA hybridization using an Oligo‐dT probe. Cell cultures were fixed using 3.7% formaldehyde (FA) for 15 min at room temperature (RT). After two PBS washes, the cells were incubated in Protease III diluted 1:30 in PBS (#322337 Advanced Cell Diagnostics) for 15 min at RT. After two more PBS washes, cells were incubated in hybridization buffer (#10369 Cepham Life Sciences) for 15 min at RT. Incubation with 5′‐Cy5‐Oligo d(T)12–18 probe (#26–4400‐02 Gene Link), diluted 1:1000 in hybridization buffer, was carried out overnight at 40°C in a humidified chamber. The following day, washes were carried out at 40°C for 5 min with 4×, 2×, and 1× SSC buffer, and with PBS. Finally, the cells were incubated with Hoechst (33 342 ThermoFisher, final concentration 1 μg/mL) and kept in PBS during imaging.

For differentiation index calculation, cells in 96‐well plates were treated with Dox for 24 h and then incubated in differentiation medium for 72 h. In the differentiated cell culture, myotubes were marked for MyHC expression using the immunohistochemistry procedure using the MF20 antibody (Table S1).

Cell cultures in 96‐well plates were incubated with differentiated or proliferating growth media. The results shown in the figures, are from a representative experiment in three biological replicates. All cellular assays were repeated three times in three biological replicates.

### 2.6 Imaging and Image Quantification

#### 2.6.1 Imaging and Analysis Workflow on the CellInsight CX7 LZR Platform

The CellInsight CX7 LZR high content screening (HCS) platform was used for imaging and image quantification using the accompanied HCS toolbox spot detector and co‐localization (ThermoFisher Scientific). Between 2000 and 12,000 nuclei objects were imaged, and each experiment was made in 3–4 biological replicates, at least twice. Cells were imaged with 405 nm (DAPI), 488 nm (YFP) filters, and per cellular assay with the following filters: imaging: TMRM with 560 nm, OPP‐555 with 560 nm, oligo‐dT‐Cy5 with 647 nm, and MF20 antibody with 647 nm.

Imaging for calculation of differentiation index was made with a 10× objective covering over 12,000 nuclei per replicate, and imaging for nuclear YFP quantification with a 20× objective, covering at least 5000 nuclei per replicate. The co‐localization toolbox was used for the quantification of differentiation index by the percentage of myonuclei without MyHC objects, and the spot detection toolbox for the YFP puncta, TMRM, OPP‐555, and oligo‐dT‐Cy5. Bulk YFP intensity was considered with a low threshold (50–150) and high threshold of puncta (650–1200). The exact threshold was adjusted per experiment; for each experiment both low and high thresholds were quantified. Analysis of both TMRM and 555‐OPP MFI was made from the perinuclear region. Oligo‐dT signal and overlap with YFP was measured from both nuclear and perinuclear regions in YFP‐positive myonuclei.

#### 2.6.2 Confocal Imaging

Imaging of single nuclei was performed with a Leica SP8 confocal microscope using a 63×/1.3 oil objective and HyD detectors, or the Dragonfly spinning disc module using a 40×/1.3 or 63×/1.3 oil immersion objective. In fixed cells, the nuclear counterstain was with DAPI (Sigma–Aldrich # D9542). In living cells, nuclear counterstain was made with Sir700‐DNA kit (Spirochrome # SC015). Imaging of DAPI was at 405 nm, YFP—488 nm, anti‐Cy5‐647, and Sir‐700 at 700 nm. Imaging settings including exposure time, laser power, excitation‐emission range, and Z‐stack steps were consistent between Ala10 and Ala16 within an experiment. Time‐lapse imaging was performed with Z‐stack acquisition for 4.20 min (1 frame per 2 s). Maximum projection of Z‐stacks was generated with Imaris. Experiments were repeated three times. The results shown in the figures are from a representative experiment.

Quantifications of confocal images were carried out in ImageJ (v1.54f).

1. Overlaying of time‐lapse images was performed using frames at 0, 130, and 260 s. Each frame was assigned a distinct RGB color, and an overlay was generated with ImageJ.

2. YFP puncta quantification was carried out with a macro in ImageJ. The YFP channel was processed with a Gaussian blur (1.5 sigma radius) “Blur” and “Despeckle” functions. Masking of the YFP puncta was applied with a constant threshold across all images within an experiment. The threshold was manually determined to match the YFP puncta (examples are shown in Figure S1A). Particles > 0.01 μm^2^ in area were considered for analysis. From each punctum, the mean fluorescence intensity (MFI), the area, and circularity were recorded. Analysis was conducted on gated myonuclei from myotubes (multinucleated) or undifferentiated cells (mononucleated cells).

3. Oligo‐dT signal analysis was carried out on gated myonuclei in multinucleated cells in ImageJ. YFP puncta and oligo‐dT analysis were carried out with a Gaussian blur of 1.0; per fluorophore, a constant threshold was used for all images (Figure S1B). The MFI and area were recorded. The overlap and correlation between YFP and oligo‐dT were assessed with the JACoP plugin [22] in ImageJ, using the M1 & M2 coefficients and the Pearson correlation.

#### 2.6.3 Transmission Electron Microscopy

Differentiated cell cultures were fixed in 1.5% glutaraldehyde in 0.1 M Sodium Cacodylate buffer for 2 h and were successively incubated in 1% Osmium Tetroxide in 0.1 M cacodylate buffer for 1 h and in 1% Uranyl Acetate in water for 1 h. The cells were then dehydrated through a series of incubations in Ethanol (70%–100%) for 90 min and embedded in Epon. The flat embedded cells were sectioned with an ultramicrotome (UC6, Leica, Vienna) using a 35° diamond knife (Diatome, Biel, Switzerland) at a nominal section thickness of 90 nm. The sections were transferred to a formvar, and carbon coated 1 × 2 mm copper slot grid and stained for 20 min with 7% uranyl acetate in water and for 10 min with lead citrate. EM images were recorded using a Tecnai 12 electron microscope (Thermo Fisher Scientific) equipped with an EAGLE 4 k × 4 k digital camera. For navigation on EM images, montages of images at 11 000× were generated using stitching software [23]. Morphology was assessed by sampling 100 nuclei on stitched EM images. For 3D reconstruction, 8 consecutive serial sections with a nominal thickness of 200 nm were manually aligned, segmented using the software program, Ais [24] and rendered as 3D isosurfaces in ChimeraX [25]. For correlative light and electron microscopy, cells were grown on a gridded μ‐Dish 35 mm plate and stained with DAPI. The living cells were imaged in an EVOS FL Digital Inverted Fluorescence Microscope (Invitrogen) equipped with a 20× objective. YFP was visualized with a GFP filter and DAPI with a UV filter. After imaging in the light microscope, the cells were processed for electron microscopy as described above. Superimposition and correlation of light and electron microscopy images were performed using Photoshop. In Photoshop, the LM image was copied as a layer into the EM image and made 50% transparent. The LM image required transformation to align with the broader scale of the EM image. This involved isotropic scaling and rotation. Alignment was facilitated by utilizing the nuclear DAPI staining alongside cell morphology.

#### 2.6.4 Holo‐Tomographic Microscopy (HTM)

Holo‐tomographic microscopy (HTM), in combination with epifluorescence, was performed on the 3D Cell‐Explorer Fluo (Nanolive, Ecublens, Switzerland) using a 60× air objective (NA = 0.8) at a wavelength of *λ* = 520 nm (Class 1 low power laser, sample exposure 0.2 mW/mm^2^) and CMOS Sony sensor, with quantum efficiency (typical) 70% (at 545 nm), dark noise (typical) 6.6 e‐, dynamic range (typical) 73.7 dB, field of view 90 × 90 × 30 μm, axial resolution 400 nm, and maximum temporal resolution 0.5 3D RI volume per second. Acquired RI images were processed with built‐in software (Nanolive). ImageJ/Fiji (https://imagej.nih.gov/) was used for the final processing and quantification. Experiments were performed in two independent experiments, and the reported results are from a representative experiment.

Nuclei texture analysis—RI images of each cell type were initially converted to the 8‐bit format using ImageJ. Subsequently, the areas, including the cell nuclei, were outlined, and extracted using a free‐hand selection tool. Following this, the texture of these selected areas was analyzed utilizing the GLCM (Gray Level Co‐occurrence Matrix) texture analysis plug‐in (version 0.4) developed by Julio E. Cabrera. This plug‐in facilitated the computation of various statistical parameters associated with the GLCM of the image, including the Inverse Difference Moment (IDM) and Entropy.

### 2.7 Statistics and Online Analysis

Statistical tests were performed in GraphPad Prism. The means of Ala10 and Ala16 were compared with a The Student's *t*‐test or one way‐Anova.

AlphaFold predictions were carried out in AlphaFold 3.0 https://alphafoldserver.com.

## 3 Results

### 3.1 Ala16‐YFP Aggregation Is Higher Than Ala10‐YFP in an Inducible Muscle Cell Model

**FIGURE 1:**
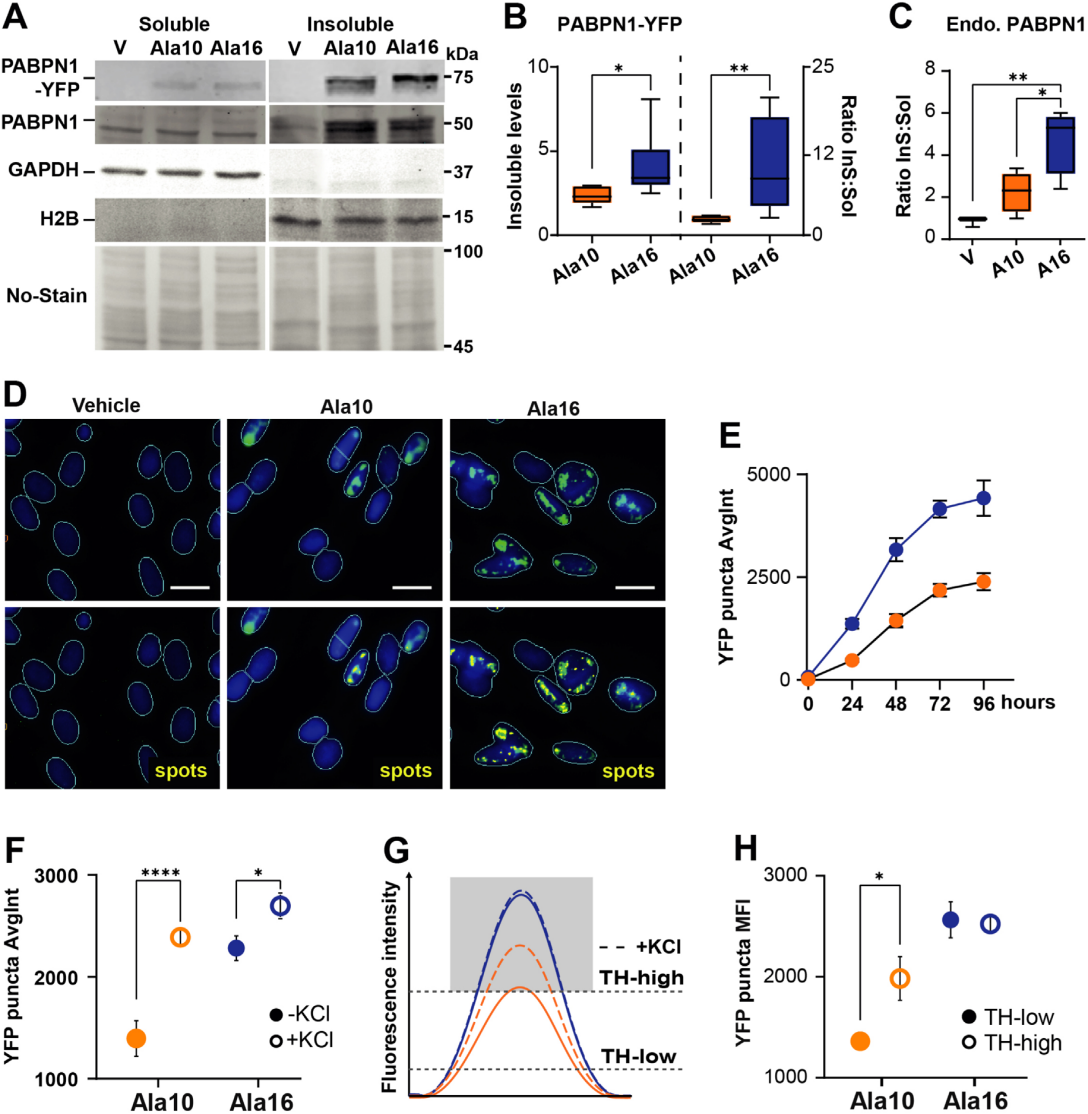
Differential aggregation and solubility of Ala10‐ and Ala16‐PABPN1 in proliferating muscle cells. Experiments were performed in cell cultures treated with Dox for 72 h. (A) A representative Western blot of soluble (sol) and insoluble (InS) fractions from vehicle (V), Ala10‐YFP and Ala16‐YFP cell cultures. PABPN1‐YFP is 75 kDa, endogenous PABPN1 is 50 kDa, GAPDH and H2B are controls for soluble and insoluble fractions, respectively. No‐Stain is a loading control. (B) Box plots of insoluble PABPN1‐YFP levels in Ala10 and Ala16 (left) or insoluble/soluble ratio (right). Expression levels were normalized to the No‐stain and the Ala10 soluble fraction. Mean and standard deviation are from six biological replicates. (C) Boxplot of endogenous (Endo.) PABPN1 insoluble/soluble ratio in vehicle (V), Ala10 and Ala16. Expression levels were normalized to No staining. Mean and standard deviation are from *N* = 4. (D–H) HCS imaging and image quantification. (D) Representative images of YFP (green) and the segmented puncta in yellow (spots) in vehicle, Ala10, and Ala16 myonuclei. The segmented nuclei are outlined in green. Scale bar is 10 μm. (E) Accumulation of YFP puncta fluorescence intensity over time. Mean and standard deviation are from *N* = 3 biological replicates, each replicate represent ~3000 cells. (F) Dot plot showing average YFP bulk intensity in Ala10 and Ala16 myonuclei with and without KCl treatment. Mean and standard deviation are from *N* = 3 biological replicates, each replicate represent ~5000 cells. (G) A schematic representation of the intensity of Ala10‐YFP (orange) or Ala16‐YFP (blue) after KCl treatment (dashed curved line) or at low or high YFP threshold. The low threshold measures the bulk YFP intensity and the high threshold measures the aggregated signal. The gray area represents the aggregated puncta. H. Dot plot shows YFP puncta intensity at low or high threshold. Mean and standard deviation are from *N* = 3 biological replicates, each replicate represent ~5000 cells. Statistical significance was evaluated using a parametric *t*‐test. *p* < 0.05 and < 0.0001 are indicated with *, ****.

To investigate differences in aggregation between a pathogenic and non‐pathogenic form in a disease‐relevant cell model, our study was performed in human muscle cells. We generated cells stably expressing the Ala10 wild‐type PABPN1 or the Ala16 extended PABPN1, both tagged with YFP (hereafter referred to as Ala10 and Ala16, respectively). Both transgenes were expressed under the tetracycline‐inducible promoter. Western blot analysis confirmed the inducible expression of both transgenes following doxycycline (Dox) treatment (Figure 1A). We assessed the difference between Ala10 and Ala16 PABPN1‐YFP aggregation by quantifying the signal from six biological replicates. Both Ala10‐YFP and Ala16‐YFP proteins accumulated in the insoluble fraction, but A16‐YFP accumulation was 1.5 times higher than A10‐YFP (Figure 1B). Additionally, the average ratio of insoluble to soluble protein levels was 3.5 times higher for A16‐YFP than for A10‐YFP (Figure 1B). We then examined whether transgene overexpression was associated with endogenous PABPN1 levels and found a higher ratio in Ala16 compared to Ala10 or in vehicle‐treated cultures (Figure 1C). Higher PABPN1 in Ala16 cells is consistent with reduced proteasome activity, which affects protein turnover [26]. This suggests that an increase in insoluble PABPN1 depletes the levels of the soluble form.

Image quantification confirmed nuclear accumulation of both Ala10 and Ala16 (Figure 1D). Consistent with Western blot analysis, YFP fluorescence intensity was 2‐fold higher in Ala16 puncta than in Ala10 puncta (Figure 1E). To verify that the YFP puncta corresponded to PABPN1 aggregates, we treated live cells with KCl prior to imaging. KCl treatment improves solubility and stabilizes aggregates of hydrophobic polypeptides [27] and has been used to detect PABPN1 aggregates in OPMD models [16, 28]. KCl treatment in the muscle cell model resulted in higher YFP intensity compared to untreated cell cultures (Figure 1F and Figure S2). Notably, the difference between KCl‐treated and untreated cells was greater in Ala10 than in Ala16, indicating that the YFP signal in Ala16 predominantly represents aggregated proteins. To identify PABPN1 aggregates without KCl treatment, we applied two thresholds during image quantification, which allowed discrimination between bulk signal at low threshold and signal in puncta at high threshold (Figure 1G). Consistent with the KCl treatment, a greater difference between low and high thresholds was found for Ala10 (Figure 1H). This analysis demonstrates that PABPN1 aggregates can be distinguished from the soluble protein puncta by image quantification, which may indicate structural differences between pathogenic and non‐pathogenic protein aggregates.

### 3.2 Structural Features Discriminate Between Non‐Pathogenic and Pathogenic PABPN1 Aggregates

The PABPN1 protein comprises a poly‐alanine stretch within the N‐terminal intrinsically disordered region (IDR), a coiled coil domain (CCD), and a C‐terminal RNA recognition motif (RRM) (Figure S3A). *Logicoil* predicts the CCD to form a tetrameric coiled coil [29], which has been shown to be critical for aggregation [8, 30]. The N‐terminal alanine stretch alone is not critical for aggregation [8], suggesting that folding of the contiguous IDR + CCD region may form a stable structure leading to aggregation. AlphaFold3 [31] prediction of the monomeric IDR + CCD predicts, with low confidence, that the alanine tract in Ala16 folds on the CCD (Figure S3B), which may be more stable than the Ala10 structure. Taking the *Logicoil* prediction of a stable CCD tetramer, AlphaFold3 predicts that the wild type (Ala10) IDR N‐terminus is parallel to the tetrameric coiled‐coil domain. However, the Ala16 expansion is consistently predicted to intercalate with the tetrameric CCD (Figure S3C). These predictions hint at a structural role for the Ala16 expansion that might lead to pathogenesis, although the mechanism is still unclear. Considering the limitations of AlphaFold for IDR prediction [32], the difference between Ala10 and Ala16 justified the investigation of structural features using puncta structure analysis.

**FIGURE 2:**
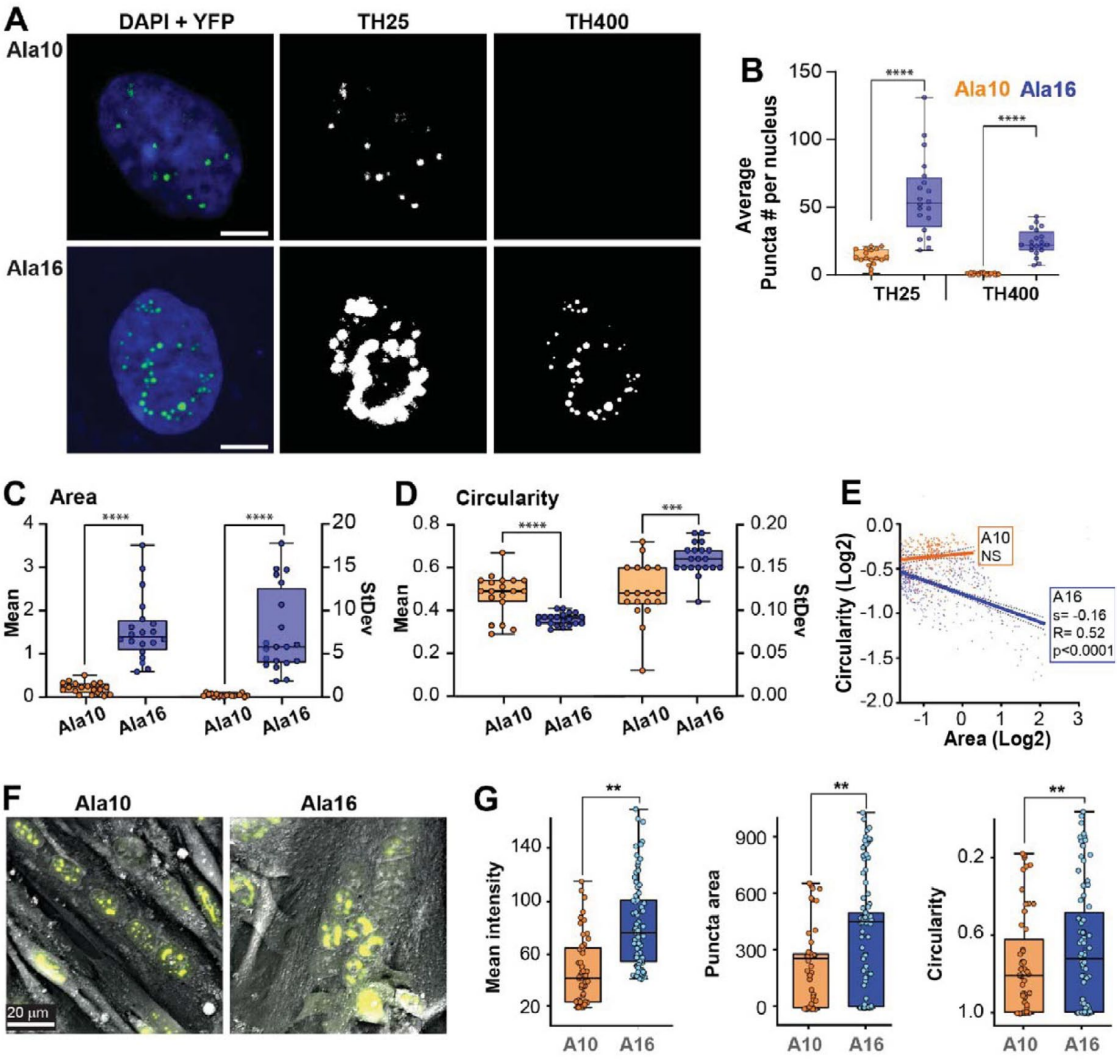
Morphological analysis of nuclear PABPN1 puncta between proliferating and differentiated myonuclei. (A–E) Experiments in proliferation conditions 72 h Dox‐treatment; (F–G) Experiments in differntion conditions 72 h Dox‐treatment. (A) Representative confocal images of Ala10‐YFP and Ala16‐YFP (in green) in a single myonucleus (DAPI counterstain is in blue) and masks of YFP puncta at low and high threshold (TH25 and TH400, respectively). The scale bar is 10 μm. (B) Boxplot showing the average number of YFP puncta per nucleus at low and high thresholds. *N* = 25 nuclei. (C–E) Analysis was performed in TH25. (C, D) Box plots show the mean and intranuclear variation of puncta area or circularity. Circles represent single nuclei. (E) A linear regression analysis of puncta [Log2 (area and circularity)], each point represents a single punctum. The significance of the F‐test, the slope (S) and the coefficient (R) are indicated. (F) Representative refractive index images of myotubes overlaid with fluorescence signal. The scale bar is 20 μm. (G) Box plots of mean YFP intensity (left), puncta area (middle), and circularity (right). Statistical significance was determined by one‐way Anova. *p* < 0.05, 0.005, 0.001 are indicated by *, ***, ****, respectively.

Structural features of PABPN1 puncta were assessed in confocal images from single nuclei (Figure 2A), and YFP puncta were segmented with a constant threshold. YFP puncta segmentation was made at two thresholds: a low threshold that visually matched the Ala10 and a high threshold that visually matched the Ala16 puncta but was too high for the Ala10 puncta (Figure 2A). A quantitative assessment of puncta segmentation confirmed the visual evaluation and showed that the average number of puncta per nucleus was significantly higher in Ala16 compared to Ala10 (Figure 2B). For comparative studies of puncta features, we considered the low threshold. The average puncta area per nucleus was 2.5‐fold larger in Ala16 than in Ala10, whereas the average circularity was smaller in Ala16 (Figure 2D). The intranuclear variability of puncta area and circularity was larger in Ala16, indicating high heterogeneity in puncta structure (Figure 2C,D). The aggregation process was assessed by the relation between puncta area and circularity. In Ala10 puncta, larger puncta remained with a similar circularity (Figure 2E). In contrast, Ala16 puncta showed a negative correlation between area and circularity (Figure 2E). Together, this suggests that Ala16 aggregation is disorganized and heterogeneous, while the Ala10 aggregates keep their shape during growth.

As the quantification of puncta structure could be affected by imaging and image processing, we verified the differences in puncta structural features using refractive index (RI) imaging combined with a fluorescence imaging platform using holo‐tomographic microscopy (HTM). This allowed us to simultaneously acquire morphological and molecular density information in selected cells (Figure 2F). Our imaging analysis showed that, per nucleus, the average fluorescence intensity in puncta was higher, size was larger, and circularity was lower in Ala16 compared to Ala10 (Figure 2G). Overall, the results from combined RI/fluorescence imaging are consistent with those obtained from confocal imaging. Taken together, nuclei in Ala16 cells were more densely packed with protein aggregates than in Ala10. This consistency across different imaging modalities and analytical techniques increases the robustness of these observations.

**FIGURE 3:**
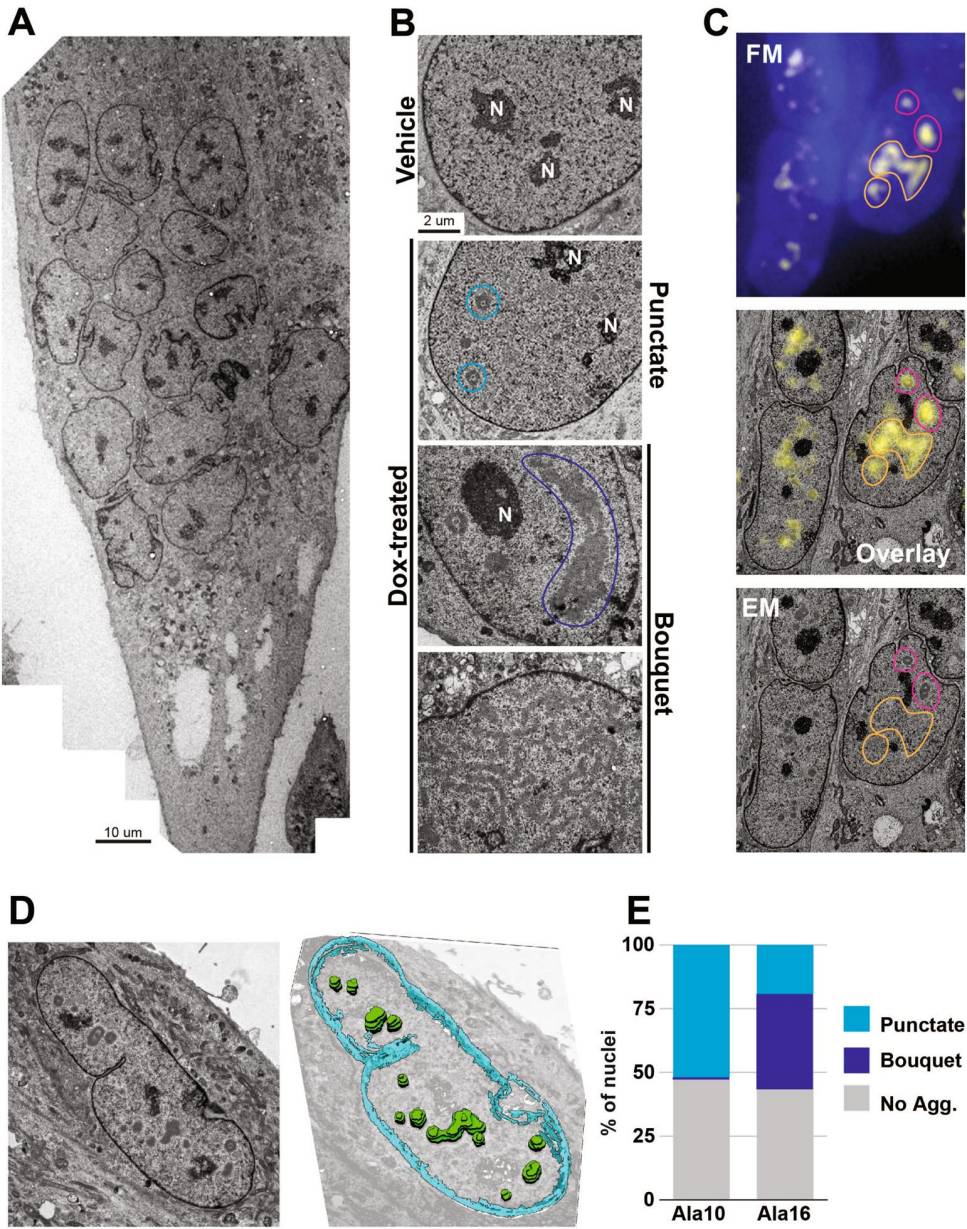
Detection of PABPN1 aggregate structures using TEM and correlative light and electron microscopy (CLEM). Differentiated cell cultures were treated with Doc for 72 h. (A) An EM image of multinucleated cell. The scale bar is 10 μm. (A) A TEM image of a multinucleated Ala16‐YFP cell. (B) TEM images of a nucleus in vehicle cell culture followed by aggregated structures: A punctate (circled in cyan) and two bouquets (circled in blue) that differ in size and shape. Nucleoli (N) are annotated. The scale bar is 2 μm. (C) Light microscopy (FM) and electron microscopy (EM) images and overlay of the same cell area. YFP signal correlating with protein aggregate structures is encircled in pink and YFP signal without correlation is encircled in orange. (D) 3D reconstruction of PABPN1 aggregates in the Ala16 cell. Left, a central section; right, a 3D reconstruction based on 8 serial sections. Aggregates are green; nuclear envelope is cyan. (E) Bar graph showing the percentage of nuclear structure (punctate or bouquet) or nuclei without aggregates in Ala10 and Ala16 myonuclei. 100 nuclei were sampled per genotype. Bar graph showing the percentage of nuclear structure (punctate or bouquet) or nuclei without aggregates in Ala10 and Ala16 myonuclei. 100 nuclei were sampled per genotype.

To verify the structure of the aggregates, we used transmission electron microscopy (TEM) to characterize PABPN1 aggregates in multinucleated cells at nanometer resolution. A stitched image of a multinucleated cell created with in‐house software [23] allowed for detailed analysis of the entire multinucleated cell (Figure 3A). In the Dox‐treated cell cultures, we observed electron‐dense structures of variable morphology. These structures, which were absent in the vehicle control myonuclei, had a different electron density than the nucleoli (Figure 3B). We classified the myonuclei based on the morphology of these electron density structures; “punctate” morphology was smaller and circular (cyan circles in Figure 3B), and ‘bouquet’ morphology was larger and not circular (encircled in blue, Figure 3B). To confirm that these novel structures corresponded to PABPN1‐YFP aggregates, we used correlative light and electron microscopy (CLEM). We correlated and overlayed fluorescence images with TEM images showing that each electron‐dense aggregate corresponded to a YFP signal (Figure 3C, circled in pink). Some YFP signals did not correspond to an electron‐dense structure because the aggregate was above or below the 90 nm thick section imaged by electron microscopy (Figure 3C, encircled in orange). Some punctate aggregates showed a toroidal architecture (Figure 3B, cyan circles). Sectioning through 3D spherical, cylindrical, or toroidal structures may result in a torus in the resulting 2D section. To determine the morphology of these aggregates, we imaged 8 consecutive 200 nm thick sections using TEM for 3D reconstruction (Figure 3D and Figure S4). This revealed that the toroidal structures represent hollow spheres of aggregated material, and that these spheres can further aggregate into larger structures.

To assess the structural differences between Ala10 and Ala16, we manually scored the aggregate structure in 90 nm thick sections of 100 nuclei. Bouquet morphology was found only in Ala16 and in 2/3 of the nuclei with electron‐dense structures (Figure 3E). In around 50% of the nuclei in both Ala10 and Ala16 myonuclei, electron‐dense structures were not found (Figure 3E), which is consistent with the 3D reconstruction analysis of PABPN1 nuclear aggregates. Taken together, confocal, holographic tomography, and TEM imaging consistently confirm the structural differences, both in area and circularity, between Ala10 and Ala16 aggregates.

### 3.3 Aggregation Features Differ Between Mononucleated and Multinucleated Muscle Cells

**FIGURE 4:**
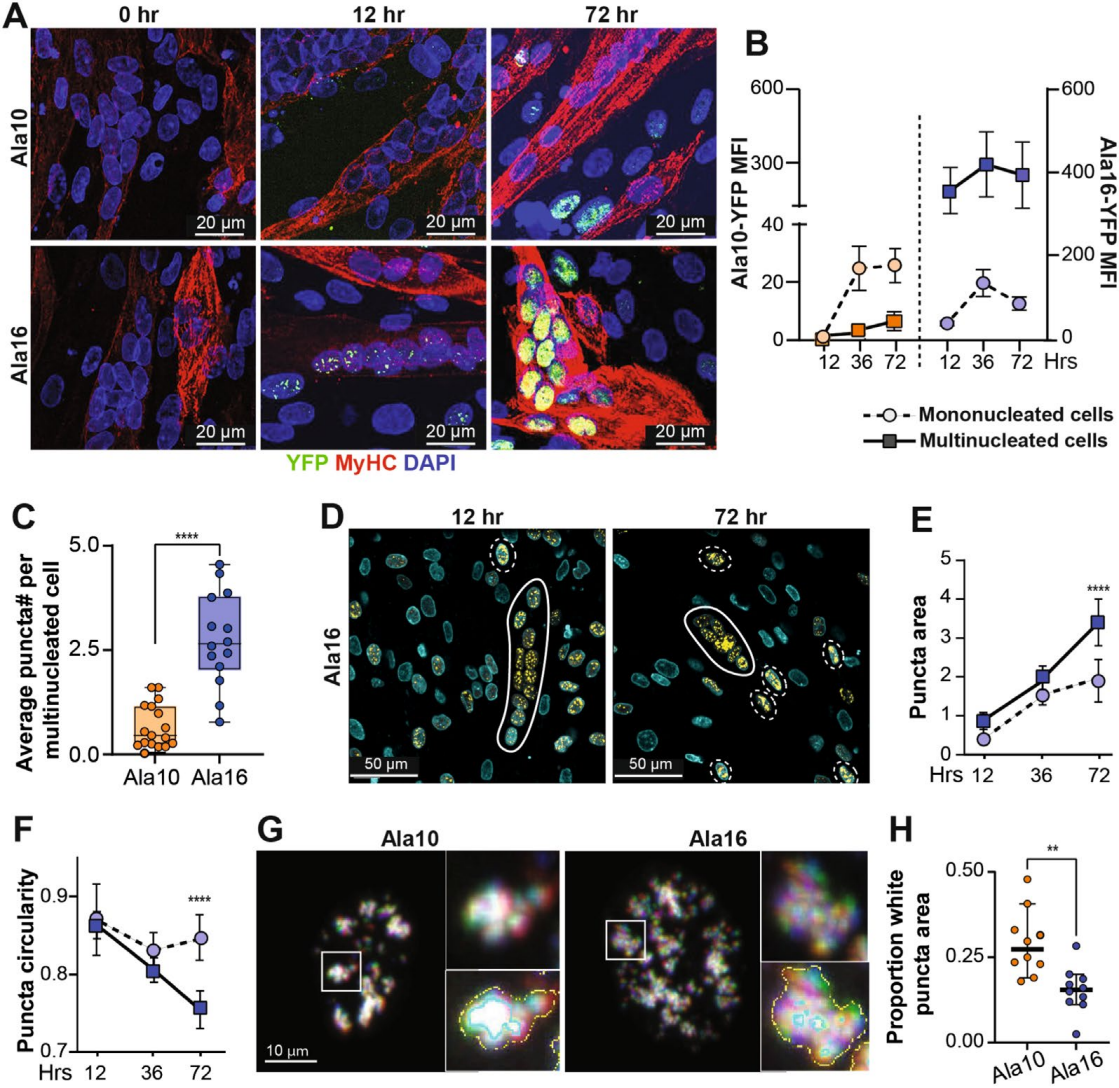
Kinetics of Ala10 and Ala16 puncta accumulation. (A) Representative confocal images of Ala10 and Ala16 cell cultures treated with Dox for 0, 12, and 72 h. Cell cultures were immunostained with anti‐MyHC, nuclei counterstained with DAPI, YFP shown in green. The scale bar is 20 μm. (B) YFP puncta MFI accumulation over time in multinucleated MyHC‐positive cells (right angle and solid line) and mononucleated cells (circle and dashed line). Mean and standard deviation are based on 100 nuclei. (C) Box plot of the average number of puncta per nucleus in multinucleated cells after 36 h of Dox treatment. Each dot represents one multinucleated cell. Mean and standard deviation are from 15 multinucleated cells. (D–F) Imaging in living cells. Nuclear counterstain was made with Sir‐700. (D) Representative confocal images of Ala16 differentiated cell cultures after 12 and 72 h of Dox treatment. Multinucleated and mononucleated objects are outlined with solid or dashed lines, respectively. Scale bar is 50 μm. (E, F) Quantification of Ala16‐YFP puncta area (E) and circularity (F) in multinucleated or mononucleated objects, denoted by continuous or dashed lines, respectively. Mean and standard deviation are from single nuclei: *N* = 41–73 mononucleated objects and *N* = 63–137 multinucleated objects. (G, H) Imaging was carried out in proliferating cell cultures starting 16 h after Dox treatment. (G) Overlay image of three frames (2‐s interval) from time‐lapse imaging. Each time frame is represented by a color (red, green, or blue); white indicates overlap of puncta. The magnification of the boxed area and the segmented white area (cyan) are shown in the right panels. Scale bar is 10 μm. (H) Dot plot of the ratio between the white area to the colored area in Ala10 and Ala16 nuclei. Standard deviation is from *N* = 10 nuclei from two experiments. Statistical significance was assessed by one‐way ANOVA. *p* < 0.005, 0.001 are indicated by **, ***, ****, respectively.

Since PABPN1 aggregation is pathogenic in muscle fibers, we investigated whether Ala10 and Ala16 aggregation differs between multinucleated and mononucleated muscle cells. In culture, high cell density and starvation stress drive muscle cell fusion into multinucleated cells, characterized by the expression of the myosin heavy chain (MyHC). Induction of Ala10 or Ala16 reduces the differentiation index compared to vehicle‐treated cells, and the myotubes in Ala16 cultures detached faster compared to Ala10 cultures (Figure S5). Therefore, to assess aggregation in differentiated cell cultures, the following protocol was used: cells were cultured in differentiation conditions for 48 h, followed by Dox treatment for 12–72 h. Cell cultures were fixed, immunolabeled with MyHC, and imaged by confocal microscopy (Figure 4A). The Ala16‐YFP signal appeared after 12 h of Dox treatment, earlier than in Ala10‐YFP cells (Figure 4A). At 72 h after Dox treatment, Ala10‐YFP fluorescence was stronger in MyHC‐negative cells compared to the nuclei within the MyHC‐positive cells (Figure 4A). To assess the statistical significance of aggregate accumulation in multinucleated versus mononucleated cells, we discriminated the multinucleated and MyHC cells from the mononucleated cells (Figure S5C) and confirmed a faster accumulation of Ala16 in differentiated cell cultures compared to Ala10 (Figure 4B). For Ala10, the fluorescence intensity is higher in mononucleated cells compared to multinucleated cells, whereas for Ala16, the fluorescence intensity is higher in multinucleated cells (Figure 4B). The difference in YFP intensity between Ala16 and Ala10 was much greater in multinucleated cells compared to mononucleated cells (Figure 4B), and the average number of puncta in multinucleated cells was also higher in Ala16 compared to Ala10 (Figure 4C).

We then compared the puncta structure of the pathogenic PABPN1 form between mononucleated and multinucleated cells. To eliminate potential image quantification artifacts due to fixation, live cell imaging was performed and the multinucleated and mononucleated objects were segmented based on nuclear density (Figure 4D). With this nuclear‐based segmentation, some mononucleated objects are potentially false positives. The puncta area was significantly larger (Figure 4E), while the circularity was smaller (Figure 4F) in multinucleated objects compared to mononucleated cells.

Next, we investigated whether puncta dynamics might contribute to the differences in puncta accumulation and structure. We assessed puncta dynamics in live cells using spinning disk imaging. Cells were imaged 16 h after Dox treatment (Video S1A,B in Ala10 and Ala16, respectively). Comparative kinetic analysis between Ala10 and Ala16 was performed in nuclei with similar YFP intensity levels. To visualize puncta dynamics, three consecutive time frames (2 s apart) were superimposed and the overlap between time frames was quantitatively assessed. An overlap between frames was colored white and indicated low puncta dynamics, whereas monochromatic puncta indicated highly dynamic puncta (Figure 4G). The proportional area of white was significantly larger in Ala10 than in Ala16 (Figure 4H), indicating that puncta dynamics were lower in Ala10 than in Ala16. The higher puncta dynamics could indicate unstructured aggregation, which is consistent with the reduced circularity in Ala16. Taken together, these results suggest that Ala16 aggregation is more affected in multinucleated cells, consistent with its pathogenicity.

### 3.4 PABPN1 Pathogenic Aggregates Affect Cell Nucleus Morphology and Cellular Function

**FIGURE 5:**
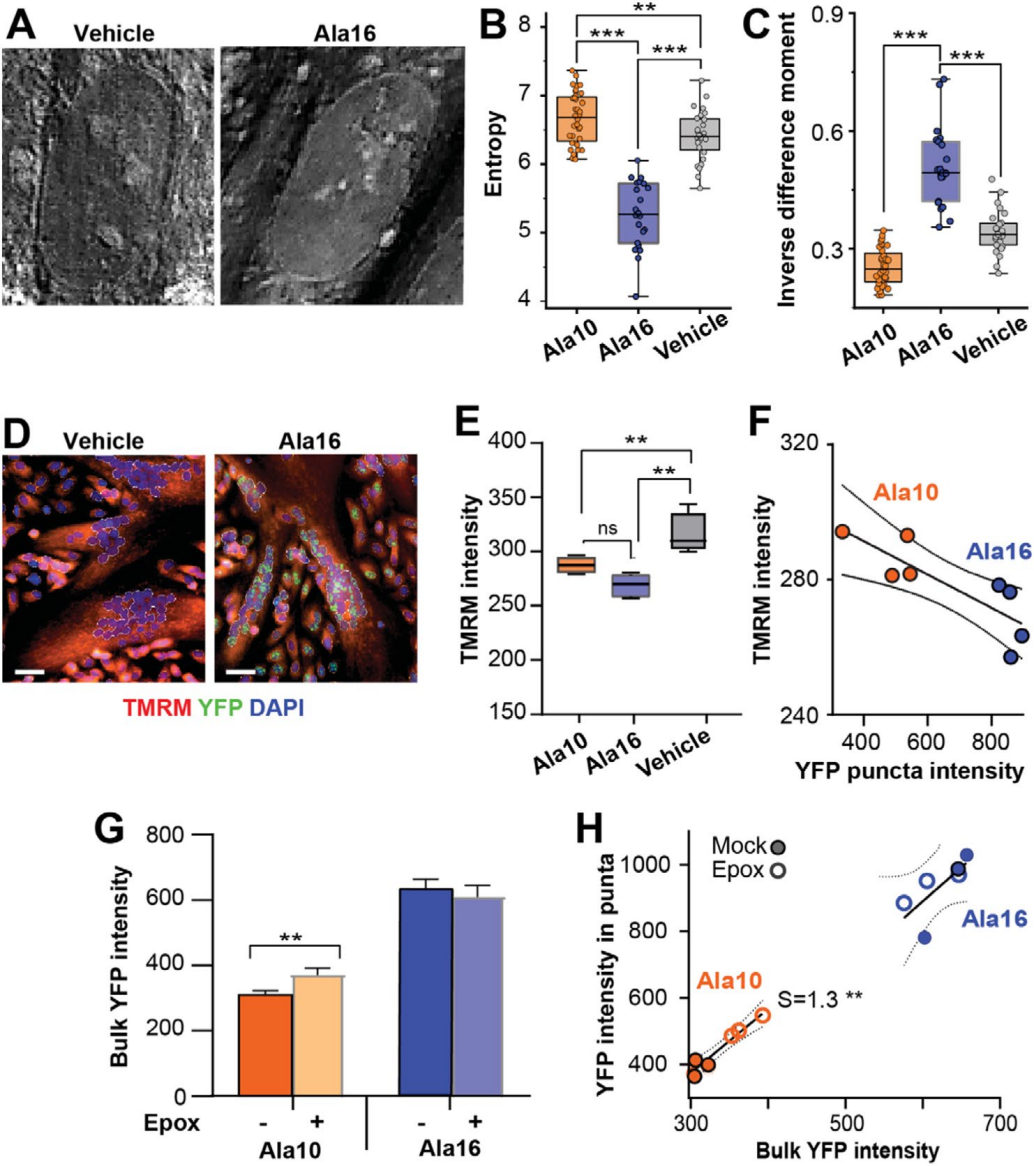
Evaluation of textural and cellular differences between Ala10 and Ala16 differentiating cell cultures. Experiments were done in Dox‐ or vehicle‐ treated cell cultures for 48 h. (A–C) Refractive index. (A) Representative images of a nucleus. (B, C) Box plots of entropy (B) or inverse difference moment (C). (C–F) TMRM assay in differentiated cell cultures. (C) Representative images of TMRM staining. Segmented multinucleated cells that were used for quantification are outlined. (E) TMRM fluorescence intensity. The scale bar is 50 μm. (F) Dot plot and correlation analysis of mean YFP intensity in puncta and mean TMRM MFI, each dot represents a biological replicate. Regression line and 95% confidence level are depicted. (G, H) Proteasome inhibition assay in differentiation conditions followed by 6 h of epoxomicin or mock treatment. (E) Bulk YFP intensity in mock or epoxomicin (Epox) treated cell cultures. (F) Dot plot and correlation analysis between mean YFP intensity and YFP intensity in puncta. The regression line and 95% confidence level are depicted, and a significant slope (S) is denoted. Statistical significance was assessed by one‐way ANOVA test. *p* < 0.01; < 0.005 or < 0.0001 are indicated by **, *** and ****, respectively. Data in (C–H) are from four biological replicates. Imaging and analysis in panels (D–H) were with the HCS platform, the data is from four biological replicates; each replicate represents the mean from ~4000 nuclei.

To assess if aggregate structure correlates with the cell nucleus morphology, we employed label‐free RI imaging, which allows textural features referring to myonuclei structure to be extracted. We imaged myonuclei in multinucleated cells (Figure 5A) and calculated entropy that measures the complexity and randomness of intensity patterns within the nuclear texture and Inverse Difference Moment (IDM) that evaluates image homogeneity [33]. In Ala16 myonuclei, entropy was significantly lower, indicating lower texture complexity than in Ala10 (Figure 5A). In contrast, higher IDM values were measured in Ala16, indicating a more uniform texture complexity (Figure 5B). The entropy and IDM values in Ala10 nuclei were close to those in the vehicle nuclei (Figure 5B,C). While our approach did not uncover the chemical nature or the mechanism behind the structural changes, the significant difference in nuclear texture measures between Ala10 and Al16 suggests that the pathogenic aggregates impact nuclear morphology.

We then investigated whether puncta intensity correlates with cellular mechanisms that are associated with PABPN1 levels, including mitochondrial activity [34], proteasomal activity [26, 35] and translational efficiency [36]. TMRM is sequestered in active mitochondria, and higher TMRM intensity indicates higher mitochondrial activity. TMRM intensity was reduced in both Ala10 and Ala16 multinucleated cells compared to vehicle‐treated cells, but did not differ between Ala10 and Ala16 (Figure 5D,E). However, TMRM intensity was not affected by YFP puncta intensity (Figure 5F). TMRM intensity was higher in differentiating cell cultures compared to proliferating conditions, but even in proliferating conditions, TMRM intensity did not differ between Ala10 and Ala16 (Figure S6A). This suggests that PABPN1 aggregation negatively affects mitochondrial activity regardless of aggregate size or differentiation condition.

Proteasome inhibition by epoxomicin treatment in differentiated cell cultures resulted in higher Ala10 YFP fluorescence, but Ala16 YFP intensity was unchanged (Figure 5G). Furthermore, YFP puncta intensity was significantly correlated in Ala10 mock‐treated differentiated and epoxomicin‐treated cell cultures, but no correlation was found in Ala16 differentiated cell cultures (Figure 5H). This suggests that proteasomal activity is impaired in Ala16 cells, in agreement with previous studies [26, 35].

**FIGURE 6:**
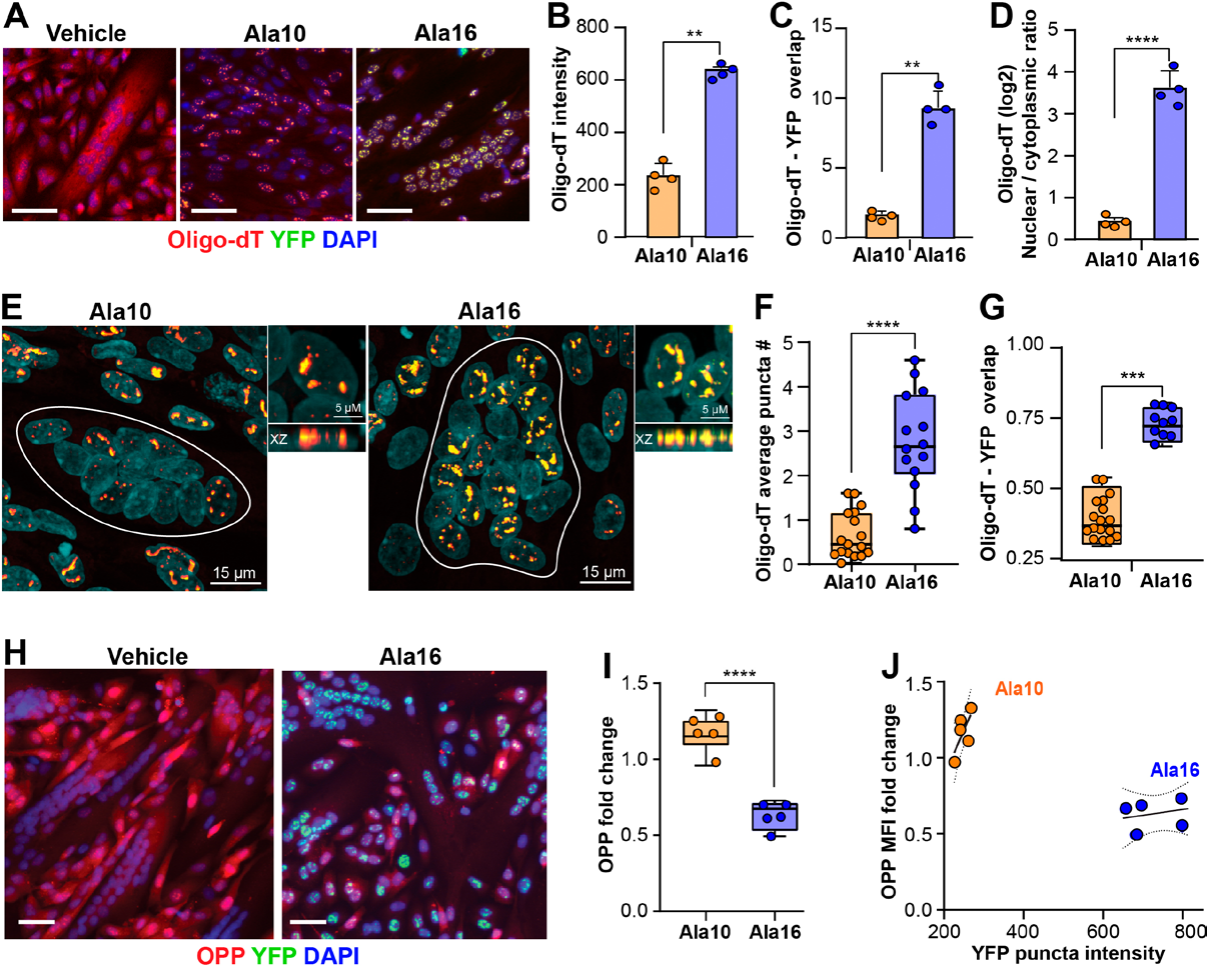
mRNA binding properties of Ala10 and Ala16 aggregates. Experiments were made in 48 h Dox‐treated cell cultures. (A) Representative images of vehicle Ala10 and Ala16 cells labeled with Oligo‐dt‐Cy5. The scale bar is 50 μm. (B–D) Bar graphs of image quantification in Ala10 and Ala16 differentiated cell cultures: (B) nuclear oligo‐dT intensity, (C), Oligo‐dT and YFP signal overlap, (D) oligo‐dT (log2[nuclear/cytoplasmic]) ratio. Averages are taken from four biological replicates. Per replicate > 1000 nuclear objects. (EC) Representative confocal images of YFP and oligo‐dT. The scale bar is 15 μm. An XY and XZ plane from Z‐stacks in Ala10 and Ala16 myonuclei. Scale bar is 5 μm. YFP‐oligo‐dT overlap is yellow. Multinucleated objects are encircled by a white line. (F–G) Analysis of multinucleated objects Oligo‐dT‐YFP overlap in confocal images. Box plots of average Oligo‐dT puncta number (F) and Oligo‐dT—YFP signal overlap (G). Means and standard deviations are from 17 and 14 multinucleated objects in Ala10 and Ala16, respectively. (H–J) Translation efficiency in fused cell cultures assessed by OPP‐Cy5 incorporation using the HCS platform. (H) Representative images of Ala16 vehicle and Dox‐treated (48 h) differentiated cell cultures labeled with OPP‐555. The scale bar is 50 μm. (I) Boxplot of OPP MFI cell cultures. MFI values indicate the proportion of vehicle cell cultures. (J) Dot plot and correlation analysis between mean YFP intensity in puncta and OPP MFI. The regression line and 95% confidence level are depicted. Means, and standard deviations are from *N* = 5. Statistical significance was assessed by one‐way ANOVA test, *p* < 0.01, < 0.005, or < 0.0001 are indicated by **, ***, ****, respectively. Images and analysis in panels (A–D) and (H–J) were made with the HCS platform.

In OPMD, mRNA is sequestered in nuclear aggregates [37], therefore, we examined mRNA co‐localization with PABPN1‐YFP signal in differentiated and proliferating cell cultures. Nuclear sequestering of mRNA was observed in both Ala10 and Ala16 cells, but not in vehicle cell cultures (Figure 6A). Quantification of oligo‐dT revealed a 3‐fold higher signal in Ala16 compared to Ala10 in differentiating cell cultures (Figure 6B). In proliferating cell cultures, the oligo‐dT signal was only 1.7‐fold higher in Ala16 compared to Ala10 (Figure S6C). The signal overlap between oligo‐dT and YFP was also significantly higher in Ala16 compared to Ala10 in both differentiated cell and proliferating cell cultures, although the correlation was higher in differentiated cell cultures (Figure 6C and Figure S6D). This suggests that higher mRNA nuclear inclusion is caused by Ala16 aggregates and is exacerbated by cell differentiation.

To verify the results obtained from HCS imaging and to investigate whether oligo‐dT co‐localizes with PABPN1 puncta, we used confocal imaging in differentiated cells. Z‐stack imaging showed co‐localization between oligo‐dT and YFP puncta (Figure 6E). The average number of oligo‐dT puncta and the overlap with YFP puncta in multinucleated cells were significantly higher for Ala16 (Figure 6F,G). The consistency of results between the two imaging platforms suggests that mRNA is entrapped in PABPN1 aggregates, which could imply limited levels of mRNA in the cytosol and thus reduced translational efficiency.

We assessed nuclear export of mRNA by the ratio of nuclear to perinuclear oligo‐dT intensity in the HCS platform and found a higher ratio in Ala16 (Figure 6D), supporting nuclear export of mRNA. Treatment with leptomycin B (LMB), a nuclear export inhibitor, showed a higher nuclear to perinuclear ratio in Ala10, indicating nuclear export (Figure S6H). However, LMB treatment had no effect in Ala16 cells (Figure S6E). This suggests impaired nuclear export in Ala16 cells. LMB treatment in differentiating cell cultures did not affect the subcellular accumulation of oligo‐dT (Figure S6E), indicating that nuclear export of the protein differs between cell culture conditions.

Last, we examined whether translation efficiency, as measured by OPP‐cy5, correlated with PABPN1 aggregation and mRNA nuclear inclusion. Translation efficiency was significantly lower in Ala16 differentiating cells compared to Ala10 cells (Figure 6H,I and Figure S6G). Translation efficiency was more affected in Ala16 differentiated cell cultures compared to proliferation conditions (Figure S6F–G). YFP puncta intensity negatively correlated with translation efficiency (Figure 6J). Taken together, our results demonstrate a strong correlation between Ala16‐PABPN1 aggregates and mRNA nuclear entrapment, which negatively affects reduced translation efficiency.

## 4 Discussion

The puzzle of why PABPN1 aggregates are not pathogenic while a short alanine expansion is has been the subject of previous studies, most of which were performed in non‐muscle cells, and the differences in aggregation between the two isoforms were limited [16, 17]. Most disease‐associated protein aggregates are found in post‐mitotic cells, such as neurons or myofibers [5]. RNA‐protein and protein–protein interaction patterns are significantly altered during cell differentiation [38]. In particular, the chaperone network system undergoes notable changes in differentiated neuronal cells, reflecting differences in the maintenance of proteostasis between proliferating and differentiated cells [39]. This suggests that studies of PABPN1 aggregates in mitotic cells masked potential differences that are relevant for differentiated muscle cells, due to cell type‐specific regulators of proteostasis [40]. Previously, we modeled PABPN1 aggregation in immortalized mouse muscle cells, where expression of PABPN1 isoforms was under the muscle actin promoter [26]. Although the transgene overexpression levels were high, aggregation was limited and PABPN1 function was not unaffected [41]. Here, we modeled PABPN1 aggregation in immortalized human muscle cells and found significant structural and dynamic differences between the non‐pathogenic and pathogenic aggregates, with a greater contrast in differentiated muscle cells compared to non‐differentiated cells. Our results highlight the critical importance of studying nuclear aggregates in cell models that closely mimic disease conditions.

Using advanced microscopy across four distinct imaging modalities, we identified micro‐ to nanoscale aggregation patterns and key differences between pathogenic and non‐pathogenic aggregates. At the nanoscale, pathogenic aggregates exhibit a unique “bouquet” structure of connected punctate units, whereas non‐pathogenic aggregates form unconnected punctae (Figure 3). At the microscale, pathogenic aggregates were larger and less circular compared to the non‐pathogenic aggregates (Figure 2). We demonstrated that structural differences between pathogenic and non‐pathogenic aggregates were more pronounced in multinucleated myotube cells than in non‐differentiated cells (Figure 4). These findings suggest that Ala16 aggregation is more pronounced in myotubes, consistent with its pathogenic nature. Previous studies in non‐muscle proliferating cells have reported limited differences between aggregates of wild‐type and expanded PABPN1 proteins, suggesting that slow protein dynamics of expanded PABPN1 correlate with higher aggregation rates [17, 26]. However, our time‐lapse imaging in differentiating cells showed slower puncta dynamics in Ala10 compared to Ala16. The reduced dynamics may be attributed to reduced stickiness, which is associated with transition from liquid‐like assemblies to gel‐like aggergates. This model will also be in line with loss of circularity in Ala16, a phenomenon commonly seen in gelation of biomolecular condensates [42]. Understanding the structure and dynamics of protein aggregates should ideally be complemented by predictive models. In the case of PABPN1, the region between the alanine stretch (or the pathogenic expansion stretch) and the coiled‐coil domain is primarily intrinsically disordered. Consequently, AlphaFold3's prediction of the monomer structure is inconclusive [32]. Nevertheless, predictions of the tetramer structure revealed that the extended alanine stretch becomes entangled with the coiled‐coil domain (CCD) in a stable configuration. Future studies should clarify how the expanded alanine stretch structure affects cell pathogenesis and investigate whether disaggregation preserves PABPN1's functional integrity.

In our cell‐based analysis, we investigated the relationship between cellular function and PABPN1 aggregates. Decreased mitochondrial activity is a hallmark of NMDs [5, 43], aging muscles, and OPMD [34, 44]. We did not find a significant difference in mitochondrial activity between cells overexpressing the wild‐type or pathogenic PABPN1, or a correlation between PABPN1 puncta and mitochondrial activity (Figure 5). However, proteasome inhibition discriminated between wild‐type PABPN1 and pathogenic aggregates. When the proteasome was inhibited using epoxomicin, we observed increased accumulation of Ala10‐YFP aggregates, indicating that these are typically degraded by the proteasome. In contrast, the same treatment had no effect on Ala16‐YFP aggregates, suggesting that either Ala16 aggregates evade proteasome recognition or proteasome function is impaired in Ala16‐expressing cells (Figure 5). This impaired proteasome activity in Ala16 cells aligns with previous studies in mouse models, which indicate that PABPN1‐mediated reduced expression of proteasomal components in OPMD models compromises proteasome function [45, 46]. Furthermore, our imaging studies show that mRNA entrapment is significantly higher in pathogenic aggregates than in non‐pathogenic aggregates (Figure 6). In OPMD, poly(A) RNA sequestration within myonuclei has been reported [37], which strengthens the relevance of our cell model. Additionally, our analysis revealed that mRNA sequestration in nuclear pathogenic aggregates correlated with reduced mRNA nuclear export and translational efficiency (Figure 6), consistent with previous studies [47].

In summary, our findings reveal structural differences between pathogenic and non‐pathogenic PABPN1 protein aggregates and their impact on muscle function. Pathogenic aggregates were larger, less circular, and more dynamic compared to non‐pathogenic aggregates. Furthermore, we demonstrated a correlation between aggregate size, mRNA nuclear entrapment and nuclear export, and translational efficiency in myotubes. Key differences in aggregate behavior between proliferating and differentiating cells highlight the importance of studying protein aggregation under disease‐relevant cellular conditions.

## Author Contributions

Conceptualization: V.R., T.H.S., A.M. Methodology: V.R., T.H.S., A.M., L.M.V., M.S. Investigation: S.D.M., E.B., V.S., M.S., V.R. Visualization: S.D.M., E.B., V.S. Supervision: V.R., T.H.S., A.M. Writing – original draft: S.D.M., E.B., V.S., V.R. Writing – review and editing: V.R., T.H.S., A.M.

## Conflicts of Interest

The authors report no competing interests.

## Supporting information


Appendix S1.

Video S1A.

Video S1B.

## Data Availability

The data that support the findings of this study are available in the Materials and Methods, Results, and Appendix S1 of this article. The imaging that supports the findings of this study is available on request from the corresponding author.
