# Supplementary material for: Polyalanine Expansion in PABPN1 Alters the Structure and Dynamics of Its Nuclear Aggregates in Differentiated Muscle Cells
Source: FASEB J. 2025 Jun 24;39(12):e70748. doi: 10.1096/fj.202501097R (PMC12186728; doi:10.1096/fj.202501097R)
Supplement: Supplementary file 1 — Appendix S1. [file FSB2-39-e70748-s001.docx]

# Supplementary data

| **Table S1. List of antibodies used for western blotting and immunofluorescence** | | | | | | | | | | |  |
| --- | --- | --- | --- | --- | --- | --- | --- | --- | --- | --- | --- |
| **A: primary antibodies** | | |  | | |  | |  | | |  |
| **Protein** | **Dilution** | | | | **Host** | | **Supplier** | | **Cat. number** |  |  |
|  | **IF** | **WB** | |  | | |  | |  |  |  |
| GAPDH | - | 1:5000 | | anti-Mouse | | | Invitrogen | | MA5-15738 |  |  |
| PABPN1 | - | 1:4000 | | anti-Rabbit | | | Homemade | |  |  |  |
| Histon2B (H2B) | - | 1:1000 | | anti-Rabbit | | | Proteintech | | 15857-1-AP |  |  |
| MyHC | 1:250 | - | | anti-Mouse | | | DSHB | | MF20 |  |  |

| **B: secondary antibodies** | | |  |  |  |  |  |
| --- | --- | --- | --- | --- | --- | --- | --- |
| **Target** | **Fluorophore** | **Dil.** | **Supplier** | | |  |  |
| Anti-mouse | Cy5 | 1:5000 | Thermo Fisher Scientific | | | |  |
| WB anti-Rb/M | 800/680 | 1:10000 | IRDye 800CW or IRDye 680RD (LI-COR) | | | |  |

# Supplementary figures

#
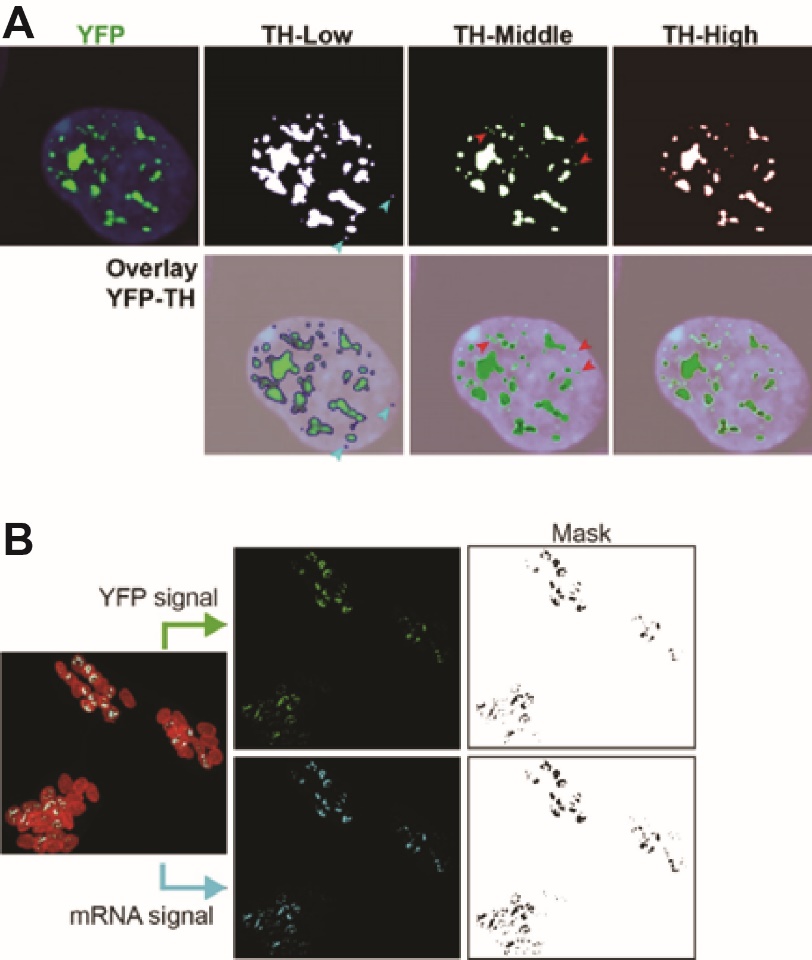


# Figure S1: Puncta segmentation. A. Threshold criteria for puncta quantification. Left is a fluorescence image (DAPI is in blue and YFP is green), and a mask of the YFP signal with increasing thresholds are to the right. An overlap between the YFP image and the threshold image is depicted in the lower panel. The cyan arrowheads point to puncta in TH-Low but are not detected in TH-Middle or TH-High. The red arrowheads point to puncta that are missing by TH-High. Most overlap was found between TH-Middle and TH-High. B. puncta quantification of YFP and mRNA signals from images of myonuclei in fused cells for quantification. The YFP and mRNA channels are isolated after which a mask is produced. These masks are used in the YFP-mRNA overlap quantification and YFP-puncta structure analysis.

#
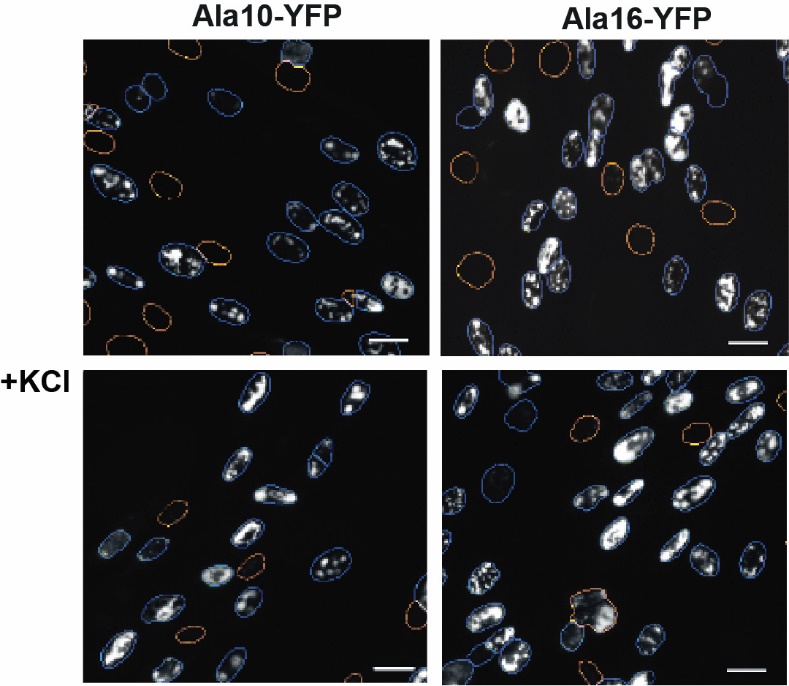


# Figure S2: Representative images of Ala10-YFP and Ala16-YFP cell culture with and without KCl treatment. Nuclei excluded from analysis are highlighted in orange, and those included are highlighted in blue. The scale bar represents 40 µm.

#
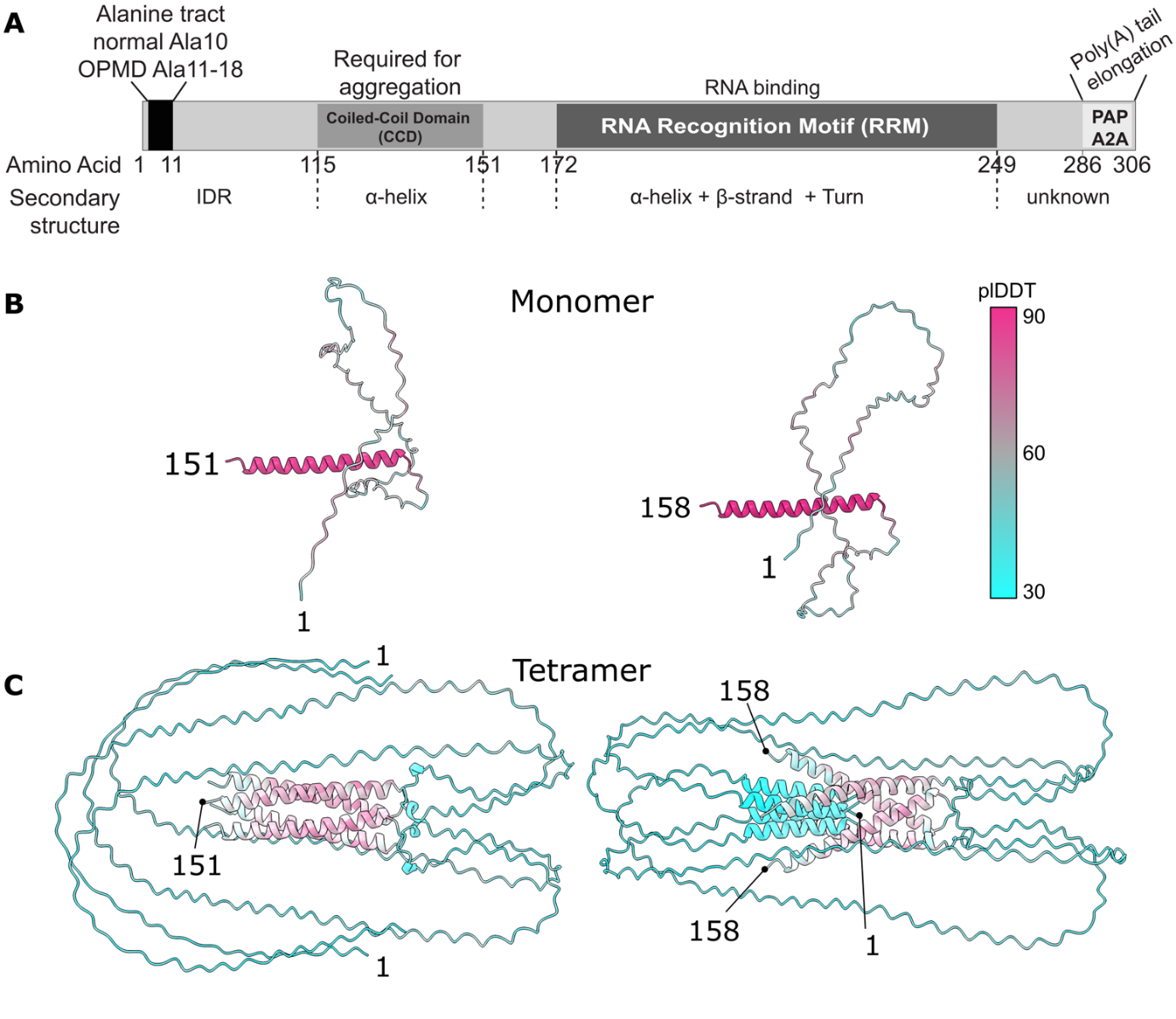


# Figure S3: Secondary structure content and Alpha-fold prediction of PABPN1 Ala10 and Ala16. A. PABPN1 regions and secondary structures. Ala10 (1-151 amino acids) and Ala16 (1-158). B. AlphaFold3 prediction of N-terminus IDR+CCD monomer. Amino-acids number at each ends are denoted. C. AlphaFold3 prediction of N-terminus IDR+CCD tetramer. The alanine-stretch is in purple. The pIDDT color scale indicate confidence in prediction. The IDR in a tetramer has a higher confidence than in a monomer, and in Ala16 higher than in Ala10.

#
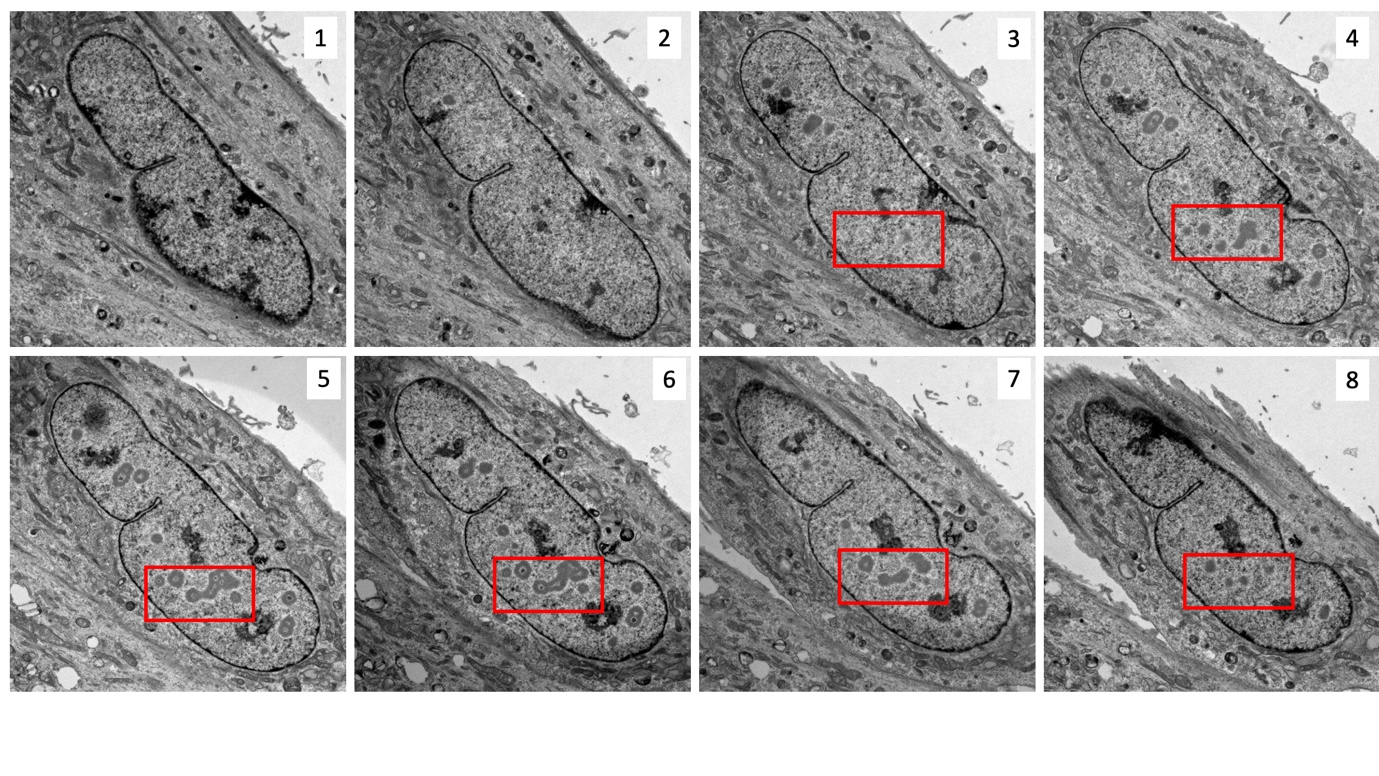


# Figure S4: Serial sections of a nucleus from an Ala16-YFP induced cell culture. The number of each sequential serial section is depicted, with a section thickness of 200 nm. The same region is indicated with a red box.

#

# Figure S5: Ala10 and Ala16 effect during muscle cell differentiation. A. Representative images of vehicle, Ala10-YFP and Ala165-YPF cell cultures after 4 or 6 days in differentiation condition. Dox treatment was in proliferating conditions, one day before differentiation condition. Differentiated cells are marked with MyHC (red) and multinucleated cells are highlighted by the segmentation blue line in the 4 days cultures. A longer incubation in differentiation condition (6 days) led to detachment of differentiated cells in Ala10 and Ala16 cells whereas in control cell cultures differentiated cells remained intact. In cells undergoing detachment MyHC intensity is high due to compression of the signal. In Ala16 cell detachment was more pronounced than in Ala10 indicating poor attachment after fusion. This observation agrees with previous studies showing that PABPN1 levels lead to cytoskeleton disorganization and poor cell mechanics properties in muscle cells. The scale bar is 50 mm. B. Differentiation index quantification in 4-days differentiating cell cultures. Average and standard deviations are from N=3. Statistical significance was assessed with a student’s t-test, p<0.05 is denoted with *. C. A confocal image of Ala16 differentiated cell culture. Differentiated cells are labeled with MyHC, myonuclei in a fused cell are encircled with a continuous line. Cells expressing MyHC but unfused are encircled with a dashed line. The scale bar is 20 µm.

#
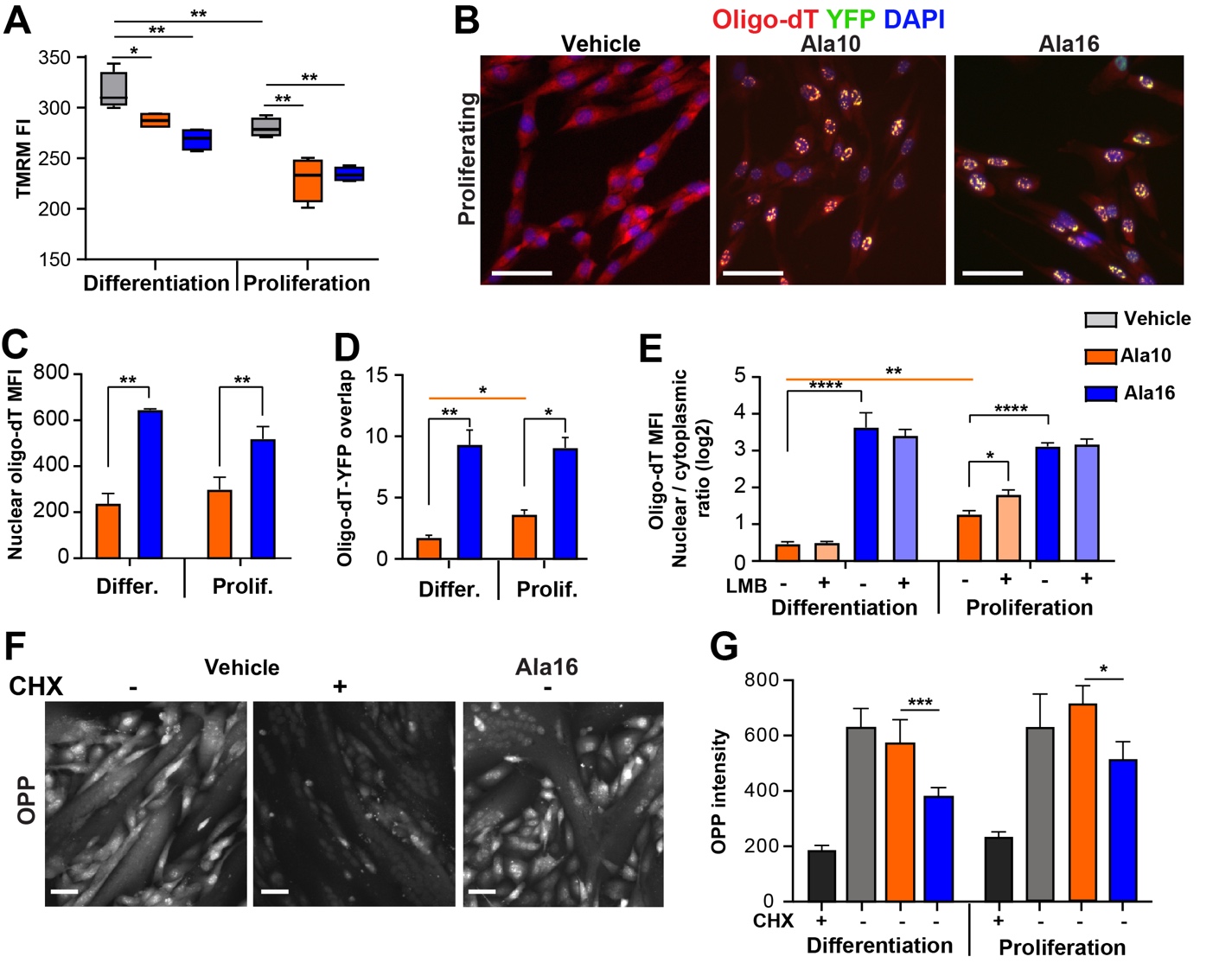
Figure S6: Activity of cellular mechanisms in proliferating and differentiating Ala10 and Ala16 cell cultures. Cell cultures were treated with Dox for three days. A. Boxplot of TMRM MFI in proliferating and differentiating cell cultures. Average and standard deviation is from N=4 biological replicates. B. Representative images of proliferating and differentiating cell culture conditions in vehicle-treated, or Dox-treated Ala10 and Ala16 cell cultures labeled with oligo-dT-Cy5. The scale bar is 50 µm. C. Nuclear oligo-dT MFI in proliferating and differentiating cell culture. D. YFP-Oligo-dT overlapping in proliferating and differentiating cell cultures. In panels C and D N=4 biological replicates. E. Nuclear to cytoplasmic Oligo-dT ratio in differentiation and proliferation cultures Mock- or LMB- treated. N=3 biological replicates. F. Representative images of vehicle-treated or Dox-treated Ala16 differentiated cell cultures labeled OPP-555. Treatment with cycloheximide (CHX) is a negative control. The scale bar is 50 µm. G. OPP MFI in proliferating and differentiating cell cultures. Average and standard deviation is from N=4 biological replicates. Statistical significance was assessed by ANOVA test, p<0.05; <0.01; <0.005 or <0.0001 are indicated by *, **, ***, ****, respectively.

# Video S1. Time-lapse imaging of puncta dynamics in Ala10 (S1A) and Ala16 (S1B) cells.
